# An automated, cost-effective and scalable, flood-and-drain based root phenotyping system for cereals

**DOI:** 10.1186/s13007-016-0135-5

**Published:** 2016-06-24

**Authors:** Michal Slota, Miroslaw Maluszynski, Iwona Szarejko

**Affiliations:** Department of Genetics, Faculty of Biology and Environmental Protection, University of Silesia, Jagiellonska 28, 40-032 Katowice, Poland

**Keywords:** Root system, Root phenotyping, Root imaging, Barley, Hydroponics

## Abstract

**Background:**

Genetic studies on the molecular mechanisms of the regulation of root growth require the characterisation of a specific root phenotype to be linked with a certain genotype. Such studies using classical labour-intensive methods are severely hindered due to the technical limitations that are associated with the impeded observation of the root system of a plant during its growth. The aim of the research presented here was to develop a reliable, cost-effective method for the analysis of a plant root phenotype that would enable the precise characterisation of the root system architecture of cereals.

**Results:**

The presented method describes a complete system for automatic supplementation and continuous sensing of culture solution supplied to plants that are grown in transparent tubes containing a solid substrate. The presented system comprises the comprehensive pipeline consisting of a modular-based and remotely-controlled plant growth system and customized imaging setup for root and shoot phenotyping. The system enables an easy extension of the experimental capacity in order to form a combined platform that is comprised of parallel modules, each holding up to 48 plants. The conducted experiments focused on the selection of the most suitable conditions for phenotyping studies in barley: an optimal size of the glass beads, diameters of the acrylic tubes, composition of a medium, and a rate of the medium flow.

**Conclusions:**

The developed system enables an efficient, accurate and highly repeatable analysis of the morphological features of the root system of cereals. Because a simple and fully-automated control system is used, the experimental conditions can easily be normalised for different species of cereals. The scalability of the module-based system allows its capacity to be adjusted in order to meet the requirements of a particular experiment.

**Electronic supplementary material:**

The online version of this article (doi:10.1186/s13007-016-0135-5) contains supplementary material, which is available to authorized users.

## Background

The availability of high throughput genotyping techniques and the rapidly increasing amount of genomic data has significantly facilitated studies in the field of plant molecular biology. The possibility of applying cost-effective genotyping tools resulted in the better characterisation of plant genomes, the enhanced spatial and temporal resolution of gene expression profiles and the identification of the genetic polymorphism that is associated with plant phenotypic traits. However, the correlation of genetic markers with agronomically relevant traits requires the precise initial characterisation of the phenotypic variability of the genotypes that are being tested. The phenotyping process is still a bottleneck in plant-related genomics studies due to the time-consuming and labour-intensive manner of the traditional methods. In order to keep up with the technological advances of modern genomics, it is necessary to create novel phenotyping methods that have an adequate precision and throughput. The recent progress in the field of plant phenotyping includes non-invasive imaging techniques, spectroscopy, image analysis methods and robotics together with high-performance computing [1]. High-throughput phenotyping platforms should offer fully controlled environmental conditions coupled with novel sensor technology in order to improve their precision and reduce the need for biological replicates.

Root phenotyping still remains the most challenging part of the analysis of plant phenotype due to the limitations that are associated with the access to the root system of soil-grown plants during their growth. Different attempts have been made to evaluate the parameters of plant root systems through the application of destructive or non-destructive methods. The selection of an appropriate method for root phenotyping affects the subsequent outcome of the analysis of root variables. The root system of a plant has a complex three-dimensional structure that can be characterised by a variety of standard traits (number of roots, root size, total root surface, root length, root density, kinetics of root growth, average root diameter, average root length, number of lateral roots etc.) as well as more composite traits (network perimeter, solidity, convex area) [2]. Thus, most quantitative genetic studies require the application of phenotyping protocols that are rapid, accurate and that provide an adequate throughput. Consequently, the proper choice of a suitable root phenotyping method should be done carefully, and special attention should be paid to its destructivity and resolution as well as to its accessibility for automated image acquisition and analysis.

The visualisation of the below-ground parts of a plant always causes technical difficulties due to the opaque character of the soil substrate. Destructive techniques of plant root phenotyping consist of the excavation of the entire root system directly from soil or from soil-filled containers in controlled environments [3]. One of the first traditional assays for studying root phenotypes in the field, was based on the construction of so-called ‘trench profiles’. These are horizontal tunnels from which the soil is completely removed in order to reveal the root systems of plants, which are then drawn from the subsequent profile walls layer by layer [4]. Most other destructive methods of plant root phenotyping are used to extract roots from soil samples that have a defined volume using excavation methods. Soil cores can be extracted manually or in a semi-automated manner in order to determine the number of root intersections, the rooting depth or the vertical root length densities or weights [4]. There have been limited attempts to standardise and automate field-based root screening assays that were aimed at increasing the throughput of such analyses [3]. One of these methods, which is termed ‘shovelomics’, is a standardised protocol for the excavation of soil cores of a given radius, gentle washing and the evaluation of primary root angle as well as the length and density classes of lateral roots using phenotyping boards [5]. However, destructive methods that involve the excavation of roots or washing the soil cores that are derived from the field are burdened with a considerable probability of an error due to the natural soil heterogeneity. It can be attributed to the heterogeneity of soil profiles together with existing physical and chemical interactions between the local components of the rhizosphere, which can strongly affect the root system architecture [6].

**Fig. 1:**
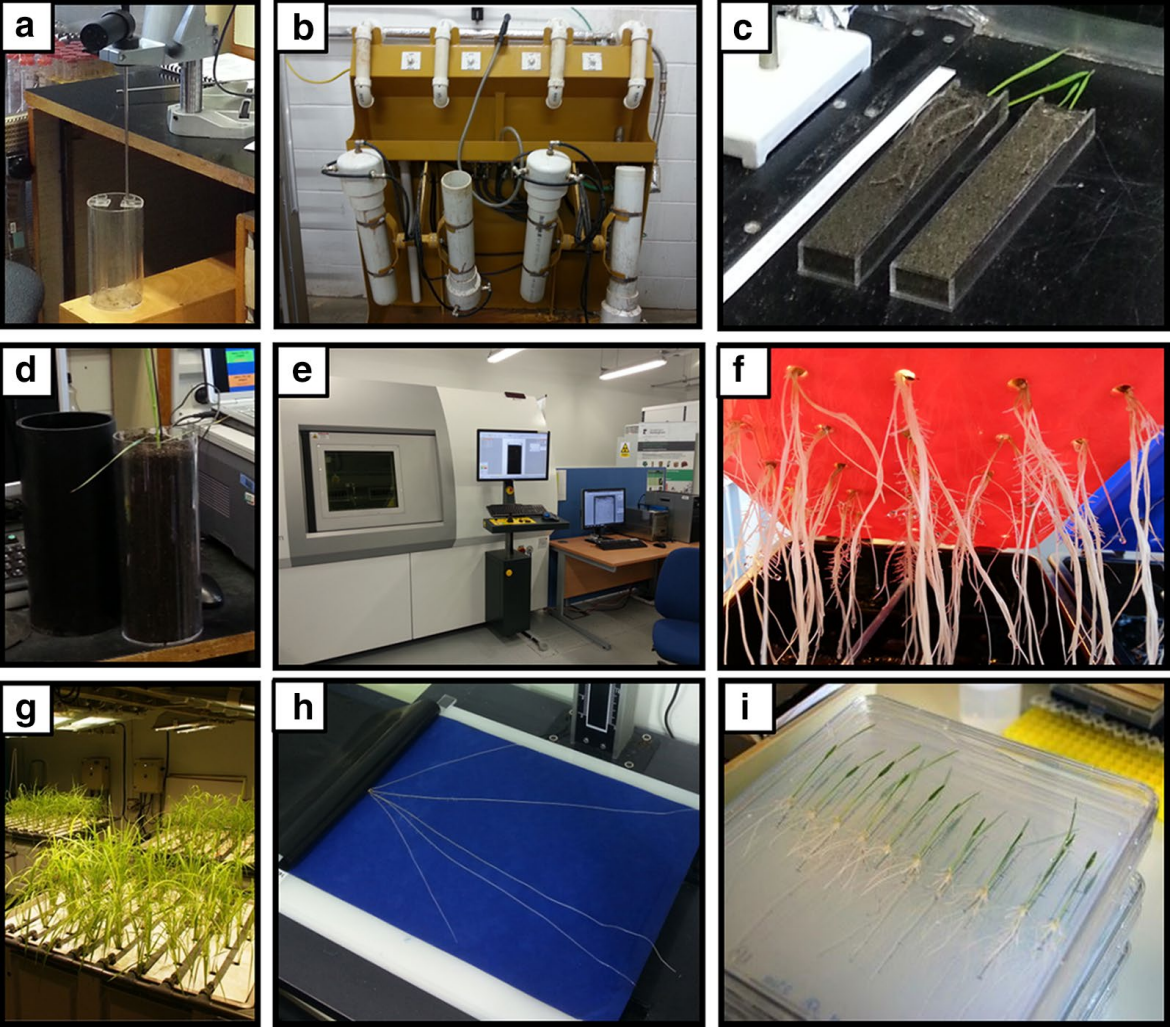
Overview of the state-of-art methods that are used in the field of plant roots phenotyping. The available methods can be characterised as soil-grown (**a**–**e**) or artificial media (**f**–**i**) systems. **a** Borescope that is used to visualise plant roots, either directly in the soil or through minirhizotron tubes. **b** Facility used for washing the soil cores derived from field. **c** Rhizoboxes that act as small rhizotrons for cereal seedlings. **d** Transparent soil column. **e** Nanotom Micro-CT scanner (Phoenix/GE Systems) that is used for the 3D reconstruction of roots in soil cores. **f** Aeroponics system for cereal seedlings. **g** Rhizoscope system that is based on hydroponics and adapted for rice. **h** Root pouches made of a filter paper. **i** Agarose plates that are used to screen seedlings. **b**, **e**, **h**—Centre for Plant Integrative Biology, University of Nottingham, **a**, **c**, **d**—Biological Research Centre, Hungarian Academy of Sciences, **f**—Department of Genetics, University of Silesia, G, **i**—Centre de Coopération Internationale en Recherche Agronomique pour le Développement. Phot. M. Slota]

Destructive root sampling methods can also be adapted for the laboratory-based screening of root traits (Fig. 1). There are a variety of assays that apply the excavation of entire root systems from soil-filled containers (e.g., pots, columns, boxes, tubes and chambers) that can be carried in a controlled environment (reviewed in [7], Fig. 1b).

A considerable effort has been made to enable the non-destructive analysis of root system architecture with special emphasis on replicability, increased throughput and automation. Non-destructive sampling methods of plant root systems generally involve the application of soil-filled rhizotrons or soil-free assays [7]. A rhizotron is a glass-walled chamber that is designed for the observation of the root growth of plants that are grown in natural or controlled conditions [7] (Fig. 1c, d). Other available methods of non-destructive root growth visualisation apply artificial media, such as hydroponics, gel chambers, filter paper-based or agar-plate systems (reviewed in [8]). The visualisation of the root system of a plant, requires a customized setup that provides favourable conditions for plant growth as well as an unhindered insight into a growing root [3]. Laboratory-based methods for root phenotyping have so far adopted a huge variety of artificial media including paper rolls [9], germination paper [10], fabric cloth [7], growth pouches [11] (Fig. 1h) and agar plates [12, 13] (Fig. 1i). These systems offer a higher degree of homogeneity in growth conditions but restrict the root growth to an unnatural 2D conformation, which limits the agronomic relevance of the analysed root parameters [14]. There have been also some successful attempts to develop a root phenotyping systems based on the application of transparent pots enabling the observation of undisturbed roots [15]. The clear-pot method was applied to evaluate the parameters of root system, such as the angle and the number of seminal roots in wheat [15]. Cultivation systems that employ a more unhampered 3D conformation of root growth consist of agar [16] or gellan gum [6] filled cylinders, hydroponics [17] (Fig. 1g) and aeroponics [18] (Fig. 1f). These systems enable the more natural growth of root systems to be visualised and are suitable for image acquisition with reduced background noise [14]. A promising attempt has been made to apply solid particles of a low refractive index that can adsorb nutrients, thus simulating the natural soil properties with favourable transparency [19]. Alternative methods for studying the 3D root architecture of soil-grown plants in a non-destructive manner are magnetic resonance imaging (MRI) and X-ray computed tomography (CT; Fig. 1e). These methods allow in situ imaging the kinetics of root growth with a high-resolution but low throughput (reviewed in [20]).

The optimal method for the detailed phenotyping of a plant root system should comprise the flexibility to test different plant species and the controllability of the experimental conditions together with the suitability for the automation of image acquisition and data processing. Although the development of high-throughput platforms for plant shoot imaging is far ahead of root phenotyping [14], numerous high-throughput root phenotyping platforms that offer a high capacity and dedicated imaging facilities have been created recently (reviewed in [21]). As a matter of a fact, root phenotyping tends to have an importance that is similar to shoot phenotyping, considering the fact that plant growth and development strongly depends on root architecture and function [3]. Root traits such as the ability to adapt to water-limited environments, resistance to soil pathogens and pests and nutrient uptake are of a great agronomic relevance [14].

The presented work describes a new method for the root phenotyping of cereals using a flood-and-drain based, remotely-controlled plant growth system that is equipped with a customised imaging setup. The system provides automatically controlled experiment conditions enabling the continuous measurements of medium parameters. An imaging setup allows for the destructive (applying WinRHIZO system) as well as non-destructive (based on RGB photography and image analysis) measurements for root and shoot phenotyping.

## Methods

### Description of the system

**Fig. 2:**
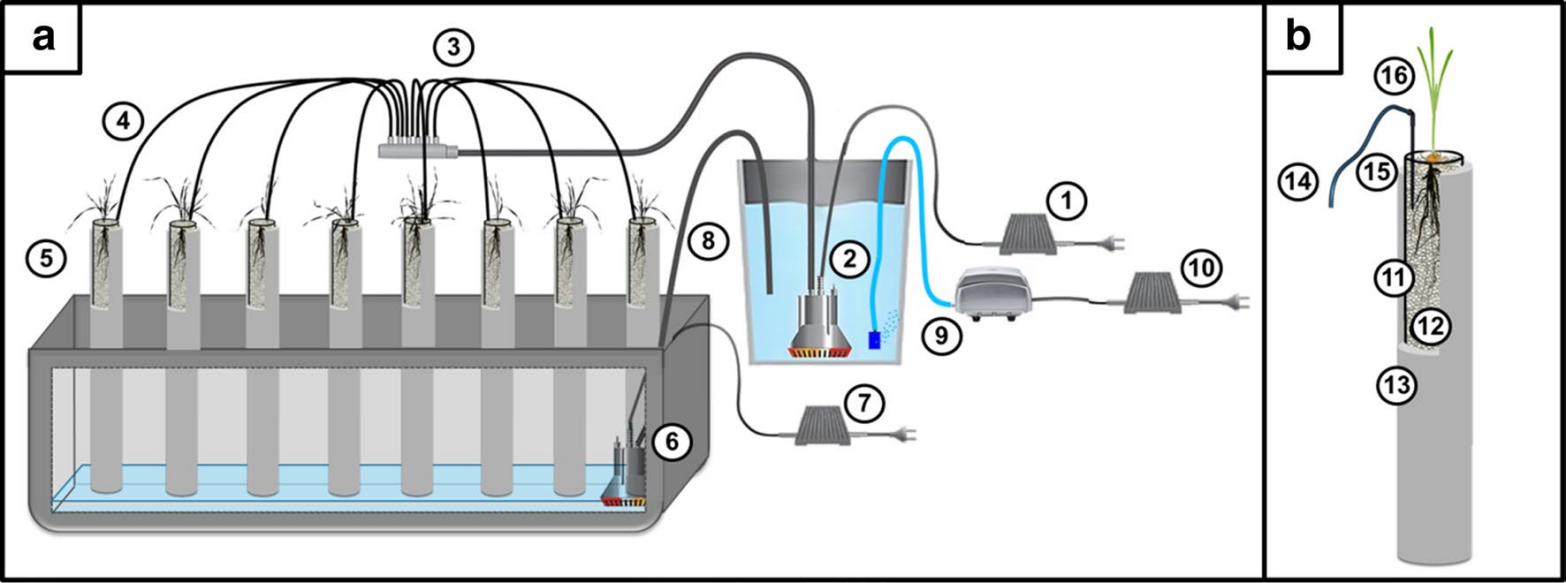
Design of the system for the automatic supply of plants with the medium. **a** Schematic overview of the watering system that is comprised of a transformer for an afferent pump (*1*), afferent water pump placed in the tank with the culture medium (*2*), distributor of 12 outlets (*3*), distribution pipes (*4*), acrylic tubes filled with a substrate (*5*), efferent pump (*6*), transformer for efferent pump (*7*), drain tubing (*8*), air pump (*9*), transformer for air pump (*10*). **b** Scheme of the medium supply system of the plant: acrylic tube (*11*), soda-lime glass beads (*12*), opaque cover tube (*13*), distribution pipe (*14*), drip-line (*15*), plant seedling (*16*)

The developed system employs an automatic drip irrigation line that is installed in transparent tubes (made of polymethyl methacrylate, 500 mm in length, 30 mm of inner diameter, 2 mm of wall thickness) filled with soda lime-glass beads (MEGAN, Gliwice, Poland; Art no. 632645102000), thereby maintaining the flood-and-drain, impeded, hydroponic conditions (Fig. 2).

Because a drip-line irrigation system is used for the supplementation of plants with the medium, precisely controlled plant growth conditions can be obtained for short-term experiments. The developed system uses an automatic drip irrigation line that is controlled remotely by a programmable logic controller (PLC). This enables the automated control of all of the system modules—water pumps, air pumps and heating devices. An afferent pump is installed in an opaque 50-l tank that is filled with a culture medium, which is dispensed via a supply tube that ends with a distributor of 12 outlets. Three types of distributors can be used interchangeably thus enabling different rates of water flow (15, 30 and 60 ml min^−1^). The solution that is applied allows the efficiency of the water flow to be adapted to the current capacity of the experiment that is being conducted. Each distributor contains 12 outlets that are connected to the branched tubing that terminates with LDPE (low-density polyethylene) drip-lines. The installed drip-lines are injected onto 50-cm-long acrylic tubes that have an inner diameter of 30 mm that are used to supplement the plants with the medium. The acrylic tubes, which have a bottom drainage opening to ensure proper draining of the medium, are placed together in a bulk container. The excess of the medium is discharged from the bulk container through an efferent pump. The transparent acrylic tubes are placed in opaque cover tubes in order to protect the roots from the light. This allows the non-invasive observation of the growth of the root system of plants via the application of a transparent substrate—soda-lime glass beads.

The PLC controller is equipped with a 36-pin Atmega328 processor, SMD version, with a 32kB Flash ROM. The controller has six electronically controlled 230 V power supply outputs. Each output can be programmed to be switched on/off depending on the measured value that is indicated by one of the linked sensors. The PLC module has an integral temperature sensor and three BNC (Bayonet Neill–Concelman) connectors that enable it to connect to any type of electrode (pH, ORP or ion-specific electrode) with a BNC socket. The setup that is demonstrated was equipped with two installed combination pH electrodes (ERH-11, Hydromet). One of the pH electrodes is inserted in the tank for the measurement of the pH of the medium, while the second is used to control the medium pH after it leaves the tubes. The PLC-controlled experiment conditions can be monitored in terms of changes in the temperature and pH at selected time intervals. The operation of the pumps can be controlled by user-defined settings or by selecting the desired threshold values for the variables that are being tested. The PLC adapter enables the afferent and efferent water pumps as well as an air pump to be programmed independently, which can therefore operate in a continuous or discontinuous module with pre-set time intervals.

**Fig. 3:**
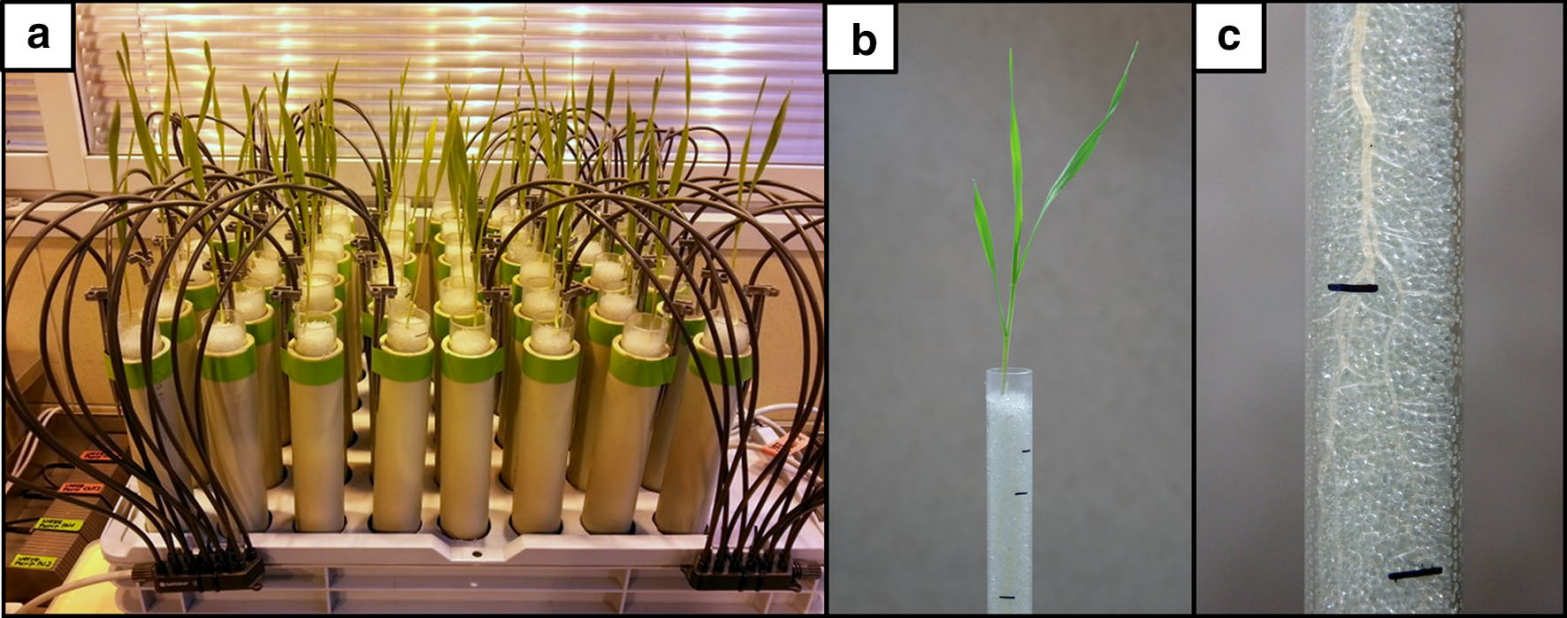
Pictures of the system setup. **a** A single module of the flood-and-drain based system that contains 48 transparent tubes, which are placed in opaque cover tubes. **b** A 14-day-old seedling of barley that was grown in an acrylic tube filled with glass beads. **c** Close-up of a fragment of the root system of barley

A single module of the growth system consists of 48 vertically set acrylic tubes that are embedded into opaque cover tubes that are mounted in the lid of a bulk container (Fig. 3a). A single seed is placed on the surface of the soda-lime glass beads that fill the acrylic tube and covered with approximately a 1 cm layer of beads (Fig. 3b). The kinetics of root growth can be observed and daily root re-growths can be marked on the surface of an acrylic tube (Fig. 3c).

The developed system was designed in such a way so as to enable an easy extension of the experimental capacity in order to form a combined platform that is comprised of parallel modules. The combined modules of the system can be controlled by the same PLC adapter using identical settings. One of the major advantages of the modular-based solution is that the system throughput can be adjusted to meet the current experimental needs.

The optimisation of the technical parameters of the system included the selection of the appropriate tubing branching system in order to achieve the appropriate rate of the supply of the medium and the proper plant growth parameters on the substrate being tested.

### Experiment setup

The appropriate amount of soda-lime glass beads is rinsed thoroughly on the sieve and autoclaved before the first use. The autoclave program was applied as follows: 121 °C, 1.1 bar, 45 min. Barley seeds were sterilized in a 5 % solution of sodium hypochlorite (Sigma, Cat. no 71696) for 15 min. Seeds were placed in sterile square plastic 120 × 120 mm Petri plates (Gosselin, Cat. no BP124-05) filled with filter paper. Seeds were kept at 4 °C for 24 h and then transferred to the incubator at 24 °C for the next 48 h. Acrylic tubes are filled with soda-lime glass beads leaving a space of approximately 5 cm at the top. The germinated seeds were transplanted into the acrylic tubes and covered with an additional portion of soda-lime glass beads leaving a space of approximately 1 cm at the top.

### Plant material and growing conditions

The plants were grown in the tubes in the greenhouse under controlled conditions—temperature 22/20 °C during the day/night, photoperiod 16/8 h and illumination of 320 μmol m^−2^ s^−1^. The plants were watered until the first leaf emerged and then supplemented with a medium for the entire duration of the experiment (14 days).

The plant material that was selected for the study consisted of:

- Spring barley (*Hordeum vulgare* L.) cultivars: ‘Diva’, ‘Karat’ and ‘Sebastian’.
- The chemically-induced mutant *rhs1.a*, derived from the barley cv. ‘Diva’. The *rhs1.a* mutant has short seminal roots and extremely short root hairs. Additionally, the *rhs1.a* mutant is characterised by dwarf phenotype: short stature, very short spikes and awns. The root length of the *rhs1.a* mutant reaches about 40 % of the parent variety at the seedling and 50 % at the spike-emergence stage and the mean diameter of the seminal roots is strongly reduced [22].
- Mutant *rhl1.a*, derived from the barley cv. ‘Karat’ after mutagenic treatment with MNU. The *rhl1.a* mutant is characterised by the lack of root hairs [22] and a different pattern of rhizodermal cells, compared to the wild-type parent cv. ‘Karat’ [23]. Both mutants, *rhs1.a* and *rhl1.a* were obtained after mutagenic treatment with *N*-methyl-*N*-nitrosourea (MNU) at the Department of Genetics, University of Silesia (Poland).

The parameters of the root system were analysed for the 14-day-old seedlings using a specialised root scanner and WinRHIZO software (Regent Instruments).

### Measured traits and variables

The system that was designed enables the intravital measurements of plant shoot traits such as shoot ‘green pixel area’ (the calculated area of the shoot canopy using a green colour filtering), convex hull area (the area of the smallest convex polygon to enclose the shoot canopy), plant height, length and the number of leaves as well as root traits—root system depth, projected root surface and number of roots at different substrate levels (Table 1). The detailed parameters of shoot and root growth can be measured in a destructive manner after completing the experiment, using manual methods (shoot and root fresh/dry weight, microscopy observation) or using specialised root scanners that are dedicated for the WinRHIZO system. The use of a PLC controller allows the desired culture medium parameters to be monitored and different time-lapse graphs of variations in the parameters to be generated.

**Table 1:** List of the measurements that are available using the system

Organ	Measurements	Destructiveness
Shoot	‘Green pixel area’ (cm^2^)	No
	Height (cm)	No
	Leaf number	No
	Shoot fresh/dry weight (g)	Yes
Root	Root system depth (cm)	No
	Projected root surface (cm^2^)	No
	Sum of the root lengths (cm)	No
	Total root length (cm)	Yes
	Projected root area (cm^2^)	Yes
	Average root diameter (mm)	Yes
	Root surface area(cm^2^)	Yes
	Root volume (cm^3^)	Yes
	Lateral root density (root cm^−1^)	Yes
	Root system fresh/dry weight (g)	Yes

### Root system scanning

The root system scanning procedure was performed using a specialised root scanner (STD4800 Scanner) coupled with WinRHIZO Pro software (Regent Instruments). After removing the plant from the acrylic tube, the roots were cut with sharp scissors to separate them and then they were placed on a waterproof tray (Regent Instruments). The roots that were being prepared for the scanning process were entirely immersed in water (see Additional file 1). The roots were positioned so as to avoid any overlapping lateral roots and to ensure a random distribution. The analysis of root system parameters was performed using the ‘Analysis’ mode. The parameters that are generated using the WinRHIZO system include the total length of the root system (cm), root system surface (cm^2^), root system volume (cm^3^) and root diameter (mm) as well as the number of tips.

The use of soda-lime glass beads also allowed the root system cleaning step to be improved for the elimination of the remaining substrate particles after the completion of the experiment. Exemplary results of the automatic processing of scan images demonstrate the minimised background noise and the elimination of culture impurities (see Additional file 2).

### Imaging setup

**Fig. 4:**
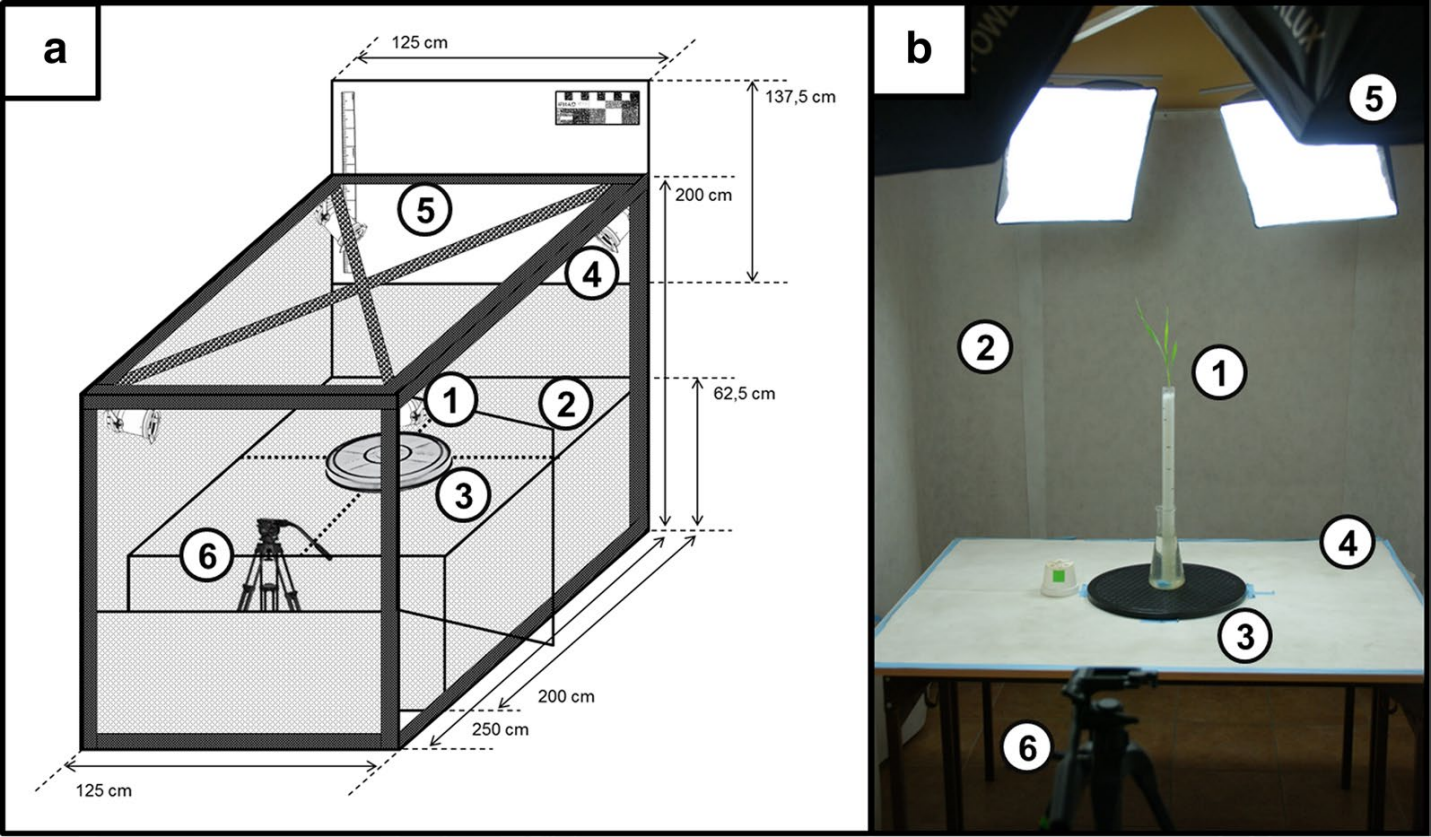
The design of the photographic light room for plant imaging. **a** Schematic representation of the instrumentation. **b** The photographic light room at the Department of Genetics, US. The object of interest (*1*) is photographed against a removable background (*2*) when placed on a turntable (*3*) located at the work-bench (*4*). The light room is equipped with four illumination soft-boxes (*5*) and a fixed camera tripod (*6*)

The non-destructive imaging of the shoot and root structure was performed in a photographic light room using a digital camera (Fig. 4). The walls of the imaging chamber were covered with an anti-reflex background for a more homogenous light saturation. The light room is equipped with four soft-boxes that are mounted at the corners, thereby providing a broad ambient light to the work areas. The daylight simulating illumination was applied with a color temperature of 5400 K (the typical color temperature of the sun during a bright day at midday). The analysis of the plant growth parameters was carried out using image segmentation methods. The analysis of shoot growth was based on the calculation of the surface of green pixels (*green pixel area*) of the plant canopy with reference to the surface of the color standard of the known dimensions. It has been shown that projected shoot area of the plants archivised on two dimensional images can be used as a parameter to predict the actual plant biomass [24]. A single or a group of acrylic tubes were photographed after assembling them in a narrow flask and placing it on a turntable. This permitted the imaging of the seedlings to be performed from different angles. The standard practice involves the imaging of four side views (0°, 90°, 180°, 270°) and a top view (optional).

### Non-destructive measurements

The visualisation of root growth during the course of the experiment can be accomplished by removing the acrylic tubes from the cover tubes after the disconnection of the watering drip lines attached to the pipe pegs which support plants from lodging. Root systems of plants growing in acrylic tubes are imaged after the staining of roots with a stirred suspension of powdered active charcoal (0.1 g l^−1^), followed by rinsing of the tubes with deionized water (according to [25]). This procedure improves significantly the contrast between the roots and unstained glass beads facilitating the process of image analysis.

### Image analysis

The analysis of the images (JPG or TIFF files) of the plants taken in photographic chamber was carried out using ImageJ [26] software. The analysis of shoot images used defined thresholds of classifiers for defining the color channel, the saturation channel and the brightness channel in the HSB (hue/saturation/brightness) colour space. The analysis of the intravital sequence of the images of plants allows to estimate a root and shoot growth kinetics (see Additional file 3).

**Fig. 5:**
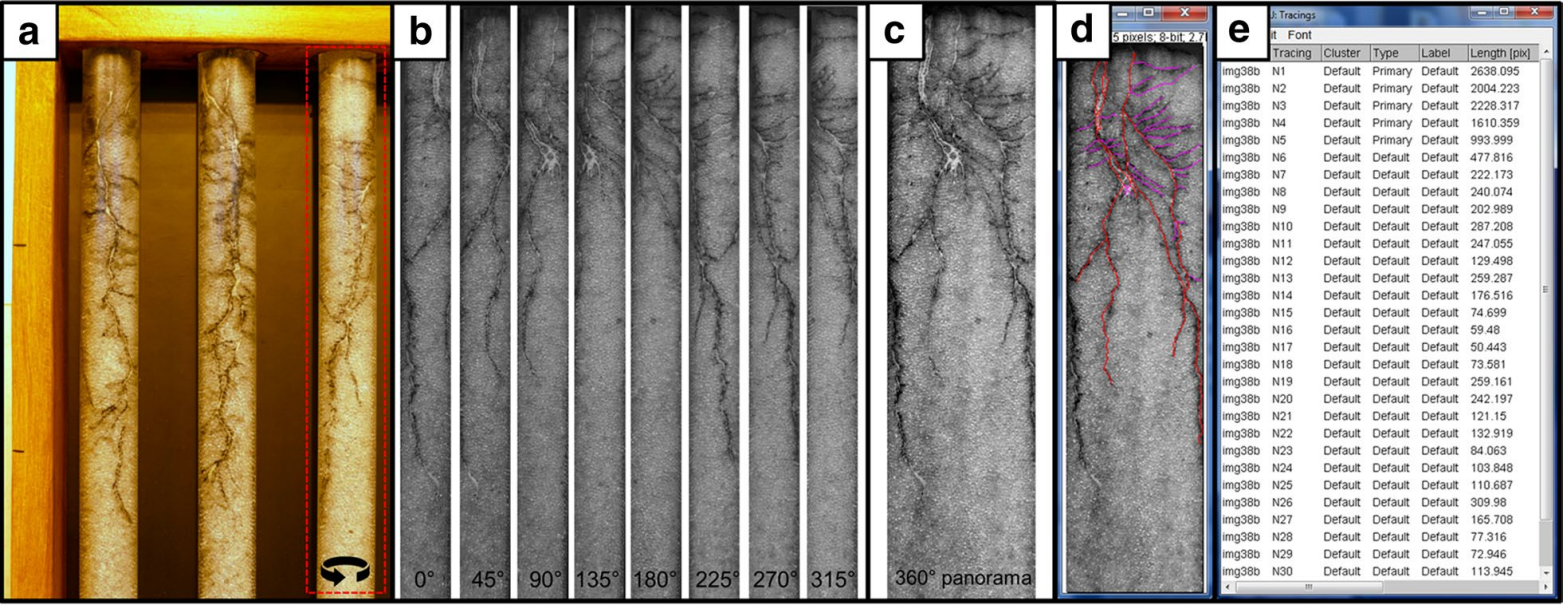
Steps of the non-invasive imaging of root systems followed by the process of image analysis. **a** Root staining with powdered active charcoal. **b** Root imaging using a RGB camera. Pictures are taken at different positions of the rotated acrylic tube with shifts of 45°. **c** The process of merging the panorama picture from the single pictures using a Panorama Maker 6 (ArcSoft) software. **d** Analysis of the root picture using a NeuronJ plugin to ImageJ software. The tracked primary roots are marked *red* whereas laterals are marked *pink*. **e** Result page of the analysis carried out using a NeuronJ plugin. Result table contains the information on assigned labels and measurements (lengths in pixels)

The analysis of the root images was carried out after the staining of roots with powdered active charcoal. Root images were taken in the photographic room using a RGB camera (Fig. 5). Pictures are taken at different positions of the rotated acrylic tube (0°, 45°, 90°, 135°, 180°, 225°, 270°, 315°). The process of merging the panorama picture from single pictures was carried out using a Panorama Maker 6 (ArcSoft) software. Analysis of the merged root picture was conducted using a NeuronJ plugin to ImageJ software. Result table generated by NeuronJ contained the information on assigned labels and measurements (lengths in pixels). A size standard of known dimensions (3–3 cm) served as the reference for the calculation of the absolute values of the root measurements.

## Results

### Selection of the size of the glass beads

**Fig. 6:**
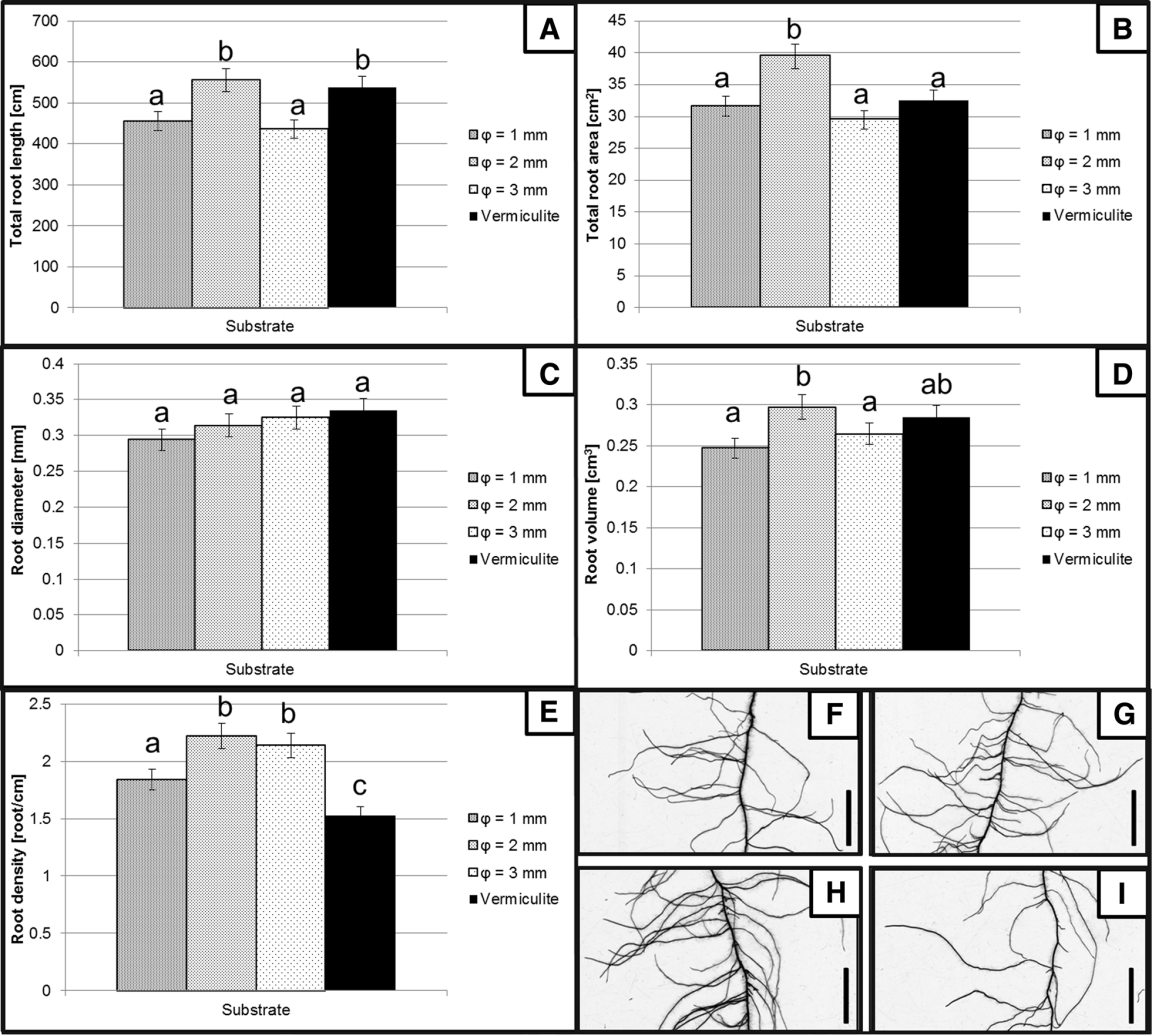
Comparison of the basic parameters of root growth of 14-day-old seedlings of barley cv. ‘Sebastian’ that were grown in the developed system using vermiculite and soda-lime glass beads of different size fractions. The measured parameters included: **A** Total root length. **B** Total surface area. **C** Average root diameter. **D** Total root volume. **E** Average root density. Fragments of the root system images of plants that were grown on the following substrates: soda-lime glass beads of 1 mm (**F**), 2 mm (**G**), 3 mm (**H**) and vermiculite (**I**). *Scale-bars* included in the **F**–**I** graphs represent 50 mm. *Different letters above the bars* indicate statistically significant differences (p < 0.05) between groups

The sizes of the soda-lime glass beads that were included in the comparative analysis consisted of three different fractions of 1, 2 and 3 mm in diameter. The comparison of the root phenotype of barley seedlings grown in acrylic tubes filled with different size-fractions of glass beads was made to previously used system that was comprised of acrylic tubes filled with vermiculite. The use of 2 mm soda-lime glass beads showed no statistical differences regarding the total length, root surface and volume and mean root diameter of 14-day-old barley seedlings in comparison to the vermiculite (Fig. 6A, B, C, D). Significant changes, i.e. the increase in lateral root density, were observed for plants that were grown in all of the sizes of the tested glass beads (Fig. 6E). The seminal roots of the plants that were grown in these types of substrates had more lateral roots per 1 cm in comparison to the vermiculite-grown plants (Fig. 6F, G, H, I). The highest lateral root density as well as the greatest length, surface and total root volume was observed in plants that were grown in the 2 mm diameter glass beads.

The substrate that was composed of 2 mm soda-lime glass beads that was finally selected as the substrate imitates the natural media compactness and has the optimal pore radius, thus providing the access of air to the roots.

### Selection of the medium

Optimisation of the physicochemical parameters of the system involved the adaptation of an appropriate medium composition for barley plants and the control of the flow of the medium in the individual tubes. As a part of the initial experiments, different variants of the composition of macro- and micro-elements in a medium were tested based on the composition of the classic Hoagland (H) medium [27] and Murashige and Skoog (MS) medium [28]. The tested variants of Hoagland medium, which is a typical culture medium for plants that are grown in hydroponic systems, differed in terms of the levels of some macronutrients—nitrogen and phosphorous as well as iron. The tested variants of Murashige and Skoog medium that is commonly used in plant in vitro cultures differed in the nutrient concentration within 1/2, 1/4 and 1/8 dilutions. The final concentrations of the selected elements in the media that were tested are shown in Table 2.

**Table 2:** Comparison of the final concentrations of the selected elements: nitrogen (N), phosphorus (P) and iron (Fe) in media that were tested

Element	Final concentration in the medium (mmol)					
	Hoagland 1	Hoagland 2	MS	1/2 MS	1/4 MS	1/8 MS
N	15	8	60.25	30.125	15.0625	7.53125
P	1	0.5	1.25	0.625	0.3125	0.15625
Fe	0.095	0.015	0.102	0.051	0.025	0.0125

The pH of the media was adjusted to ~5.9–6.1° using 1 N NaOH

**Fig. 7:**
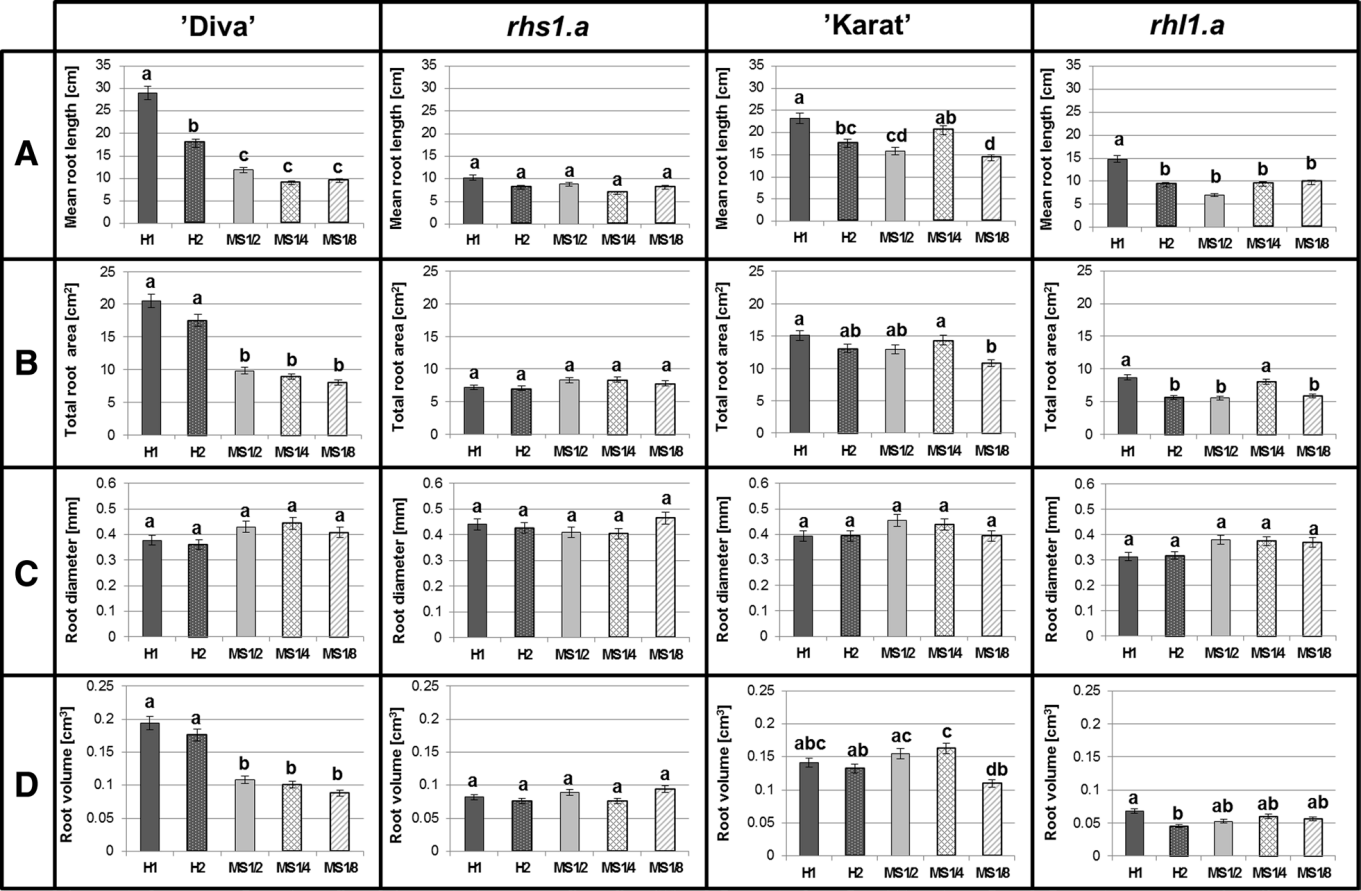
Comparison of the root system parameters of 14-day-old barley seedlings of ‘Diva’ and ‘Karat’ cultivars as well as the *rhl1.a* and *rhs1.a* mutants that were tested using different types of media. The media that were tested were composed of the Hoagland medium (H1) and its modification with a reduced concentration of nitrogen and phosphorus (H2) and the Murashige & Skoog (MS) media concentration at 1/2, 1/4 and 1/8. The parameters that were analysed included the mean root length (**A**), total root surface area (**B**) average root diameter (**C**) and total root volume (**D**). *Different letters above the bars* indicate statistically significant differences (p < 0.05) between groups

The plant material that was used for the selection of the optimal composition of culture medium comprised the seedlings of the spring barley cultivars ‘Diva’ and ‘Karat’ and their mutants—*rhs1.a* and *rhl.1.a*, respectively. Most of the measured parameters (root length, root system area and root volume) presented higher values in the case of barley plants of cv. ‘Diva’ which were supplemented with the H1 and H2 medium in comparison to those that were supplemented with MS media. In the case of barley cv. ‘Karat’, a mean length of the primary root was the only parameter that differed between the tested variant H1 of the Hoagland’s medium and H2 and all the concentrations of MS medium. A *rhs1.a* mutant with strongly reduced root system presented no statistical differences in the root parameters among the tested variants of the media. A *rhl1.a* mutant exhibited significantly higher mean length of the primary roots as well as increased total root area when supplemented with H1 medium. The parameter of the mean diameter of a root did not differ significantly between the cultivars grown on tested media. The use of H1 medium allowed for the best discrimination of the root system parameters between the parental genotypes and the mutants (cv. ‘Diva’ vs. *rhs1.a* and ‘Karat’ vs. *rhl1.a*) that were tested (Fig. 7, see Additional file 4).

### Selection of the diameter of acrylic tubes

**Fig. 8:**
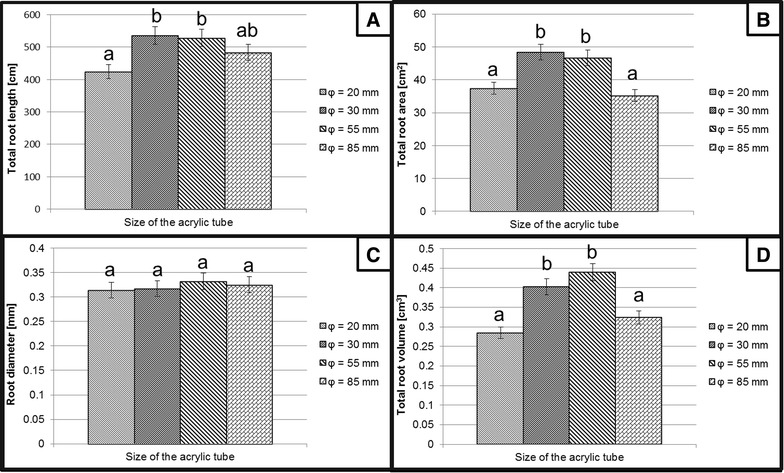
Comparison of the basic parameters of root growth of 14-day-old barley seedlings cv. ‘Sebastian’ that were grown in the developed flood-and-drain based system using different diameters of the acrylic tubes. The measured parameters included: **A** Total root length. **B** Total surface area. **C** Average root diameter. **D** Total root volume. *Different letters above the bars* indicate statistically significant differences (p < 0.05) between groups

The selection of the most appropriate diameter of the acrylic tubes was carried out after testing the following series of inner diameter: 20, 30, 55, 85 mm. All tubes were made of 2 mm thick polymethyl methacrylate and were 500 mm long. The comparison of the basic root parameters of barley seedlings grown individually in tubes was made (Fig. 8). The best root growth parameters regarding the total length, surface and volume of the root system were observed for the tubes of the diameter 30 mm and 55 mm. No differences were found in the mean diameter of the roots grown in all of the tubes tested. The tubes with the inner diameter of 30 mm were selected for further testing as they occupied less space than the 55 mm tubes and were simpler to use in non-destructive imaging.

### Comparison of destructive and non-destructive imaging methods

**Fig. 9:**
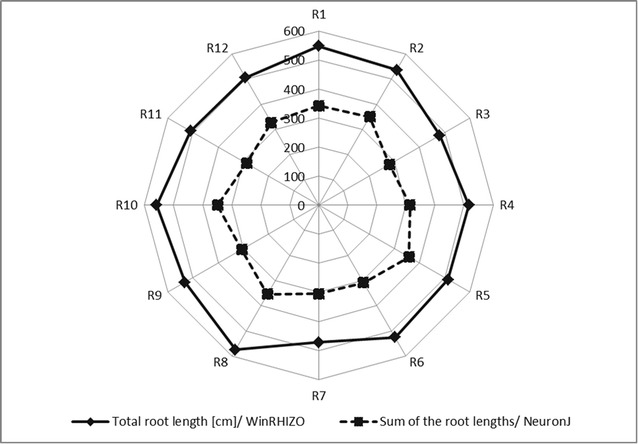
A radar chart presenting the correspondence between the total root length calculated using WinRHIZO software (a *solid line*) and a sum of the root lengths obtained using a NeuronJ plugin for ImageJ (a *dashed line*)

The correspondence between a destructive (WinRHIZO scans) and non-destructive (RGB root imaging) methods for root phenotyping using a described system was tested. A parameter of a sum of the root lengths measured using a NeuronJ plugin for ImageJ proved to be a good indicator of total root length calculated using WinRHIZO software. This parameter reached an average of 62 % of the value determined by the destructive scanning method while maintaining a constant ratio between the biological replicates (Fig. 9).

### Adjusting the medium flow

**Fig. 10:**
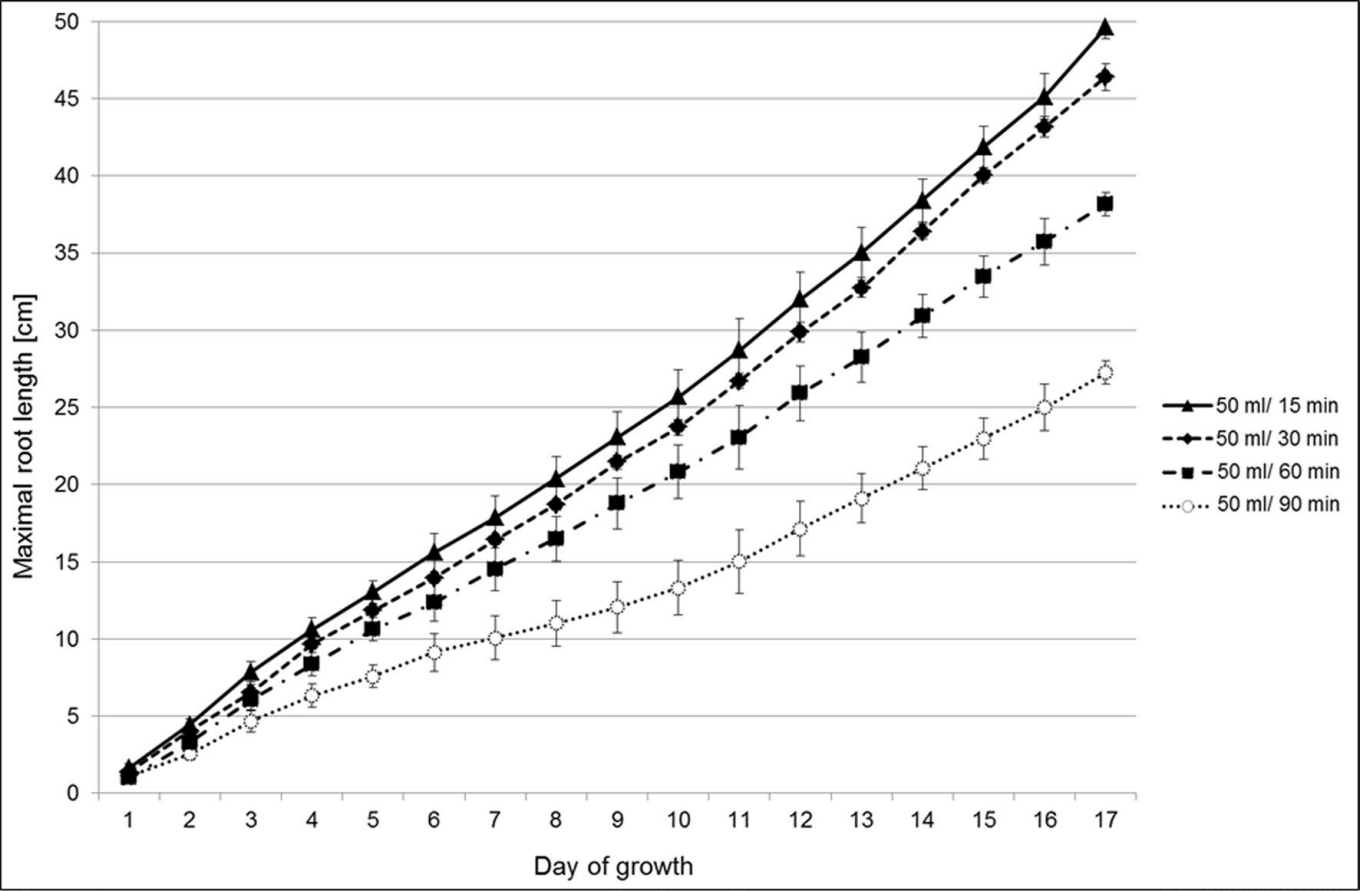
The kinetics of root growth of barley seedlings cv. ‘Sebastian’ under different medium flow rates. The maximal root lengths of plants were measured daily through the transparent acrylic tube surface during the 14-day experiment. The following medium flow rates were tested: 50 ml per 15, 30, 60 and 90 min

In the course of the optimising experiments, 16 mm diameter opaque LDPE (low-density polyethylene) tubes were selected for the optimal flow rate of the medium into the tubes filled with glass beads. The frequencies of 1-min watering programmes that were tested comprised a range of pause intervals from 15 to 90 min. Among the tested variants of supplementation, the rate of medium flow of 50 ml per plant per 15 min appeared to sustain the best kinetics of root growth. Barley seedlings that were supplemented in that manner were characterised by the greatest maximal root length with daily root re-growths of 2–2.5 cm (Fig. 10). The adjusted watering frequency also enabled the appropriate draining of the excess of the medium in order to avoid the occurrence of anaerobic conditions.

## Discussion

It has been previously demonstrated that the use of hydroponic systems that include a solid substrate for the simulation of the mechanical impedance of natural soil can lead to significant alterations of root and whole plant growth compared to classical hydroponics. The initial selection of suitable substrate for impeded hydroponics is crucial. An appropriate solid substrate used for the hydroponic cultures shall imitate some of the properties of natural soil including the mechanical impedance and porosity. Unlike the natural soil, artificial substrates should be characterized by an easy maintaining of sterility and the lack of the interactions with the components of culture solution [29]. The most basic artificial substrates applied in hydroponic studies are quartz gravel and sand [30]. Other substrates used in these studies include manufactured expanded clay balls [31], vermiculite [32, 33], perlite [34], glass-beads [35] and rockwool [33, 36]. It has been shown that the texture of growth substrate and its hardness can strongly affect the architecture of root systems [37]. The application of coarse silica beads as a growth substrate resulted in a lower resistance to root growth of rice in comparison to finer beads and sand [37]. Soda lime glass-beads selected for our study are chemically inert, autoclavable and provide an adequate mechanical impedance for root growth.

In our study, the initial choice of the tested substrate was made based on previous experiments that were carried out using a Rhizoscope platform in rice [35]. The 1,5 mm diameter glass beads that were tested in Rhizoscope system exhibited the greatest mechanical impedance for growing rice roots as the effective pore radius was smaller in comparison to the size of a root tip. The use of the larger bead sizes of about 5 mm in diameter as a substrate for root growth in rice showed no statistical difference in comparison to hydroponics [21]. In our study the measured characteristics of the root system of 14-day-old barley seedlings were improved in comparison to the unimpeded conditions of the classical hydroponics. The differences in root morphology and exudation between maize plants grown in the glass-bead impeded and unimpeded hydroponics have been demonstrated at 12th and 16th day of culture [38].

The testing of appropriate size of the glass beads carried out in the presented study indicated that the fraction of 2 mm diameter proved to be most applicable in the experiments on spring barley. Barley seedlings exhibited the highest lateral root density as well as the greatest length, surface and total root volume when grown in impeded hydroponics conditions supported with glass beads of 2 mm diameter. A modified root branching can be regarded as a trait of a high agronomic importance. It has been demonstrated that increased lateral root density correlate with the greater water and nutrient uptake [45, 46]. A reduced primary root elongation and increased lateral root density, has been observed in plants exposed to a number of abiotic stresses, and can be considered as a beneficial morphogenic response leading to the avoidance of contaminated soil patches [47].

The composition of the nutrient solution which is circulated through the acrylic tubes filled with glass beads was optimized within the framework of conducted studies. The application of classical Hoagland’s medium, widely used for plant hydroponics cultures have proved to be most suitable for the root phenotyping experiments carried out on barley. The use of aerated Hoagland’s solution appeared to provide the most optimal condition for plant growth, enabling the best discrimination of the root system parameters between the parental genotypes and the mutants with affected root phenotypes that were analysed. The concentration of nitrogen and phosphorus in the selected nutrient solution was relatively high in comparison to medium used for rice by Courtois et al. [35]. The reason of this difference can be attributed to the continuous circulation of the nutrient solution applied in that system compared to the flood-and-drain cycles proposed in the presented study.

The medium circulation intervals selected for the described system consist of 1 min supplementation cycle followed by a 15 min draining period. This rate of a medium flow appeared to sustain good kinetics of root growth of barley seedlings. The drainage/pumping cycles depend on the size of the culture solution vessels and the design of the setup. Other authors has previously tested a 20 min pump/20 min drain cycles for wheat and barley plants grown in modified hydroponics conditions employing a polycarbonate plastic fragments as a source of mechanical impedance for plant roots [38]. Other studies related to the salinity tolerance carried out on wheat successfully applied a supplementation cycles occurring every 30 min to a complete filling of growth chamber with a culture solution for the hydroponic system composed of PVC tubes filled with cylindrical black poly-carbonate pellets [29].

The selection of the size of the acrylic tubes was carried out with the following rationale behind: the maintenance of desired parameters of plant growth during the course of the experiment and the suitability for the subsequent non-invasive imaging of the root system. The choice of the tubes with the inner diameter of 30 mm was driven by the evidence of obtaining a steady kinetics of root growth together with a high proportion of the visible roots. The use of considerably narrow tubes enabled the sequential imaging of the stained root systems at different angles which facilitated the process of the image segmentation for non-destructive imaging. That allowed visualizing the kinetics of root growth at selected time points during the experiment. The correspondence between the data generated using this non-destructive imaging and destructive measurements (WinRHIZO scans) was constant for different genotypes tested. A parameter of a sum of the root lengths proved to be a good indicator of total root length calculated using WinRHIZO software. In comparison to other methods [29, 35, 38], by using a transparent substrate and advanced image processing methods, the developed system enables the ‘real-time’ phenotyping of root systems during the course of the seedling growth.

In the presented study the root system of 14-day-old barley seedlings was analysed. The application of measurements of the root traits at seedling stage as a direct proxy of a root performance of adult cereal plants has been a subject of the broad discussion. The main difference between the root systems of a seedling and the adult cereal plant is the lack of adventitious roots at the early growth stages. In addition, there is a strong dependence of a seedling root growth on the endosperm composition [39]. Nevertheless, there are numerous reports which demonstrate the sufficient association between the seedling root vigour and the adult plant root phenotype in the field. The correlation between the angle of seedling root and a depth of rooting system of the adult plant has been shown in maize, rice and sorghum [15]. In wheat, a more vertical spread of seminal roots and a higher number of seminal roots during the seedling stage have been associated with a more compact root system possessing more roots at deeper soil layers in adult plants [15]. The angle of the emergence of seminal roots of wheat seedlings have been linked to a better performance of adult plants in the field during drought [40]. The seedling root traits that correlate with a better field performance in rice comprise the thicker and longer roots [41]. Significant correlations have been also demonstrated for root traits of maize seedlings cultured in hydroponic system and the adult plants grown in the field [42]. The overlapping QTLs which influence root traits in hydroponics and field conditions have been found [43]. Similarly, studies in tetraploid wheat carried out at different environments demonstrated that 75 % of all QTL clusters for early root vigour overlapped with QTL density peaks for grain weight and/or grain yield in field conditions [44].

## Conclusions

The aim of the study was to develop a fully automated and easy-to-use system for the phenotyping of roots of cereal species for which the conditions of DWC (deep water culture) hydroponics are not favourable. The system employs an automated drip irrigation lines enabling the supplementation of the culture solution to individual plant grown in transparent tubes filled with soda-lime glass beads. The remotely-controlled experiment setup enables the continuous measurements (in automated manner) of the parameters of the culture solution, such as temperature, pH, redox potential and the concentration of specific ions. The operation of the system can be perpetual (as in classical hydroponics), periodical (based on pre-set time intervals) or irregular (initiated by the changes of the medium parameters). The flow of the medium can be easily adjusted in terms of the supplementation time, volume and periodicity. The presented results indicate the most favorable conditions for the experiments performed for barley in terms of the composition of culture solution, the type of the substrate and the supplementation programmes. A customized imaging setup designed for the system is adjusted for high quality root and shoot phenotyping. It consists of the destructive (applying WinRHIZO system) as well as non-destructive (based on RGB photography and image analysis) measurements accessibility. The construction of the growth system provides an easy access to growing roots which strongly facilitates the non-invasive observations of root growth kinetics. The obtained data suggest that the analyses of the images of root systems captured in a non-destructive manner can be a good indicator of root characteristics calculated using a more precise, destructive measurements. The system provides highly homogenous growth conditions for 48 plants in one module. The module-based construction of the system allows for the easy adjustment of the experiment throughput to the actual requirements. Each system module can be controlled by a single or a shared PLC controller which allows expanding the capacity of the experiment. It enables an easy extension of the experiment throughput by establishing a combined platform that is comprised of parallel modules. The presented system can be applicable in the wide range of research related to plant mineral nutrition and screening for tolerance to biotic and abiotic stresses. The developed method can also facilitate the analyzes of the rhizosphere-microbial interactions and the assessment of the role of substrate texture on root system architecture. It should be noted, however, that the applicability of the proposed assay is limited to the observations root system at seedling and young plant stage. The axenic nature of the applied artificial substrate and the lack of its inner heterogeneity may also hinder the application of the method as a simple proxy of natural soil systems.

The proposed method combines the advantages of high controllability of the plant growth conditions in the automated manner together with the accessibility for the high quality imaging using the adapted root and shoot phenotyping setup. That makes a developed flood-and-drain system adequate for performing short-term experiments on cereal seedlings with high reproducibility and ease of use.

## Additional files

10.1186/s13007-016-0135-5 The process of scanning the root systems. Analysis can be performed in a non-destructive (**A**) or destructive (**B**) manner. Root scanning setup use the Epson Perfection V700 Photo coupled with WinRHIZO System (Regent Instrument). A high-resolution flatbed scanner (1) has the integrated TPU (Transparency Unit) in the lid (2). The WinRHIZO system accessories consist of a scanner positioning system (3) and a translucent waterproof tray (4). When the roots of a living plant are scanned (5) the remaining part of the root system is submerged in tap water in a petri dish (6).
10.1186/s13007-016-0135-5. The output of the scanning of the root systems. **A.** A six-day-old seedling of barley scanned as a whole. **B**. Complete root system of a 14-day-old barley seedling (cv. Sebastian).
10.1186/s13007-016-0135-5 Illustration of the outcome of the image segmentation process of a barley seedling grown in the system. Shoot (**A**) and root (**B**) photographs were taken with a digital camera against a dark homogeneous background.
10.1186/s13007-016-0135-5 Amount of compounds used for the preparation of Hoagland’s medium No. 2 Basal Salt Mixture. The pH of the medium was adjusted to ~ 5,9-6,1° using 1 N NaOH.

## Abbreviations

CT: computer tomography
HSB: hue/saturation/brightness
LDPE: low-density polyethylene
MRI: magnetic resonance imaging
MS: Murashige & Skoog medium
PLC: programmable logic controller
PVC: polyvinyl chloride
RGB: red/green/blue
