# Supplementary figures and images for: An automated, cost-effective and scalable, flood-and-drain based root phenotyping system for cereals
Source: Plant Methods. 2016 Jun 24;12:34. doi: 10.1186/s13007-016-0135-5 (PMC4919847; doi:10.1186/s13007-016-0135-5)

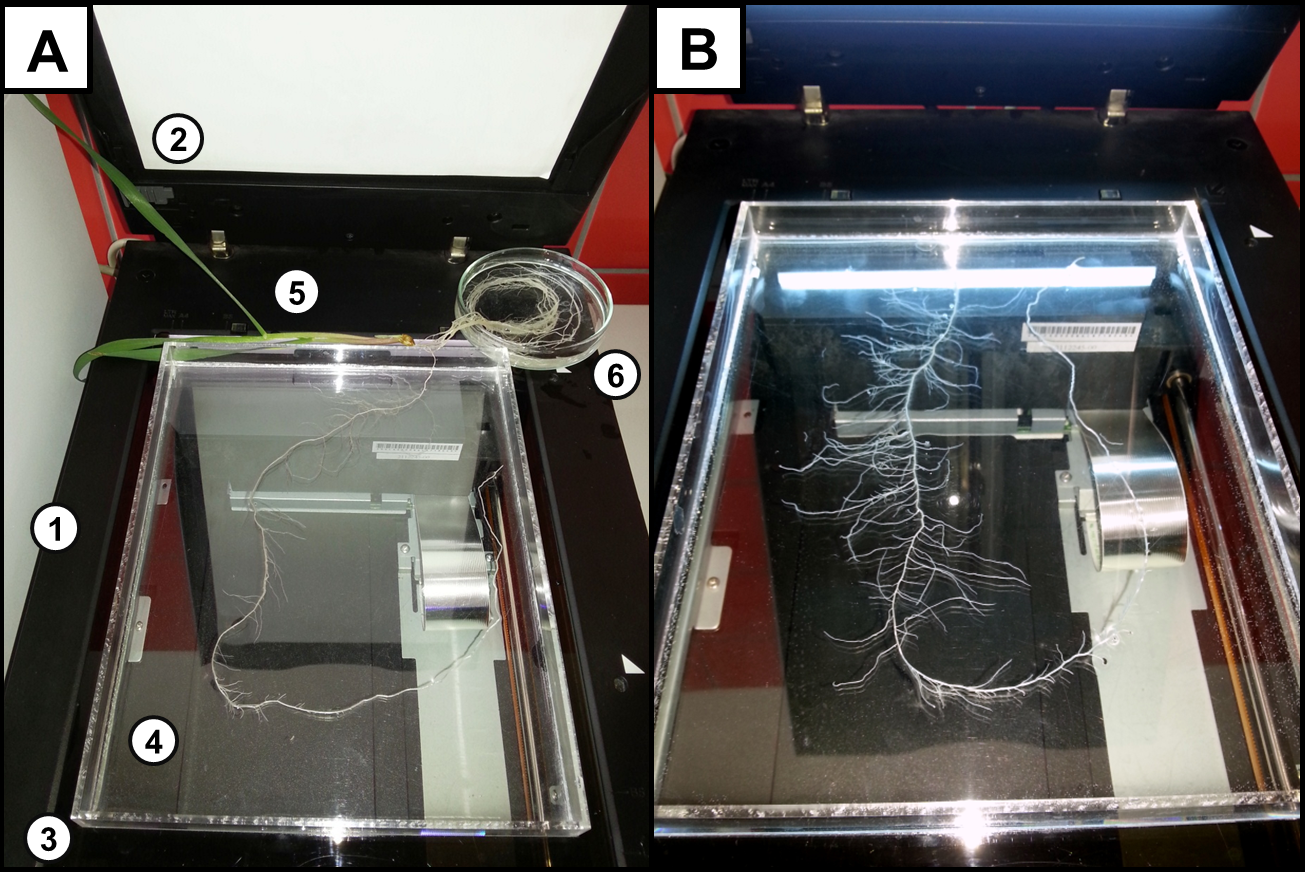

Supplement: Supplementary file 1 — 10.1186/s13007-016-0135-5 The process of scanning the root systems. Analysis can be performed in a non-destructive (A) or destructive (B) manner. Root scanning setup use the Epson Perfection V700 Photo coupled with WinRHIZO System (Regent Instrument). A high-resolution flatbed scanner (1) has the integrated TPU (Transparency Unit) in the lid (2). The WinRHIZO system accessories consist of a scanner positioning system (3) and a translucent waterproof tray (4). When the roots of a living plant are scanned (5) the remaining part of the root system is submerged in tap water in a petri dish (6). [file 13007_2016_135_MOESM1_ESM.png]

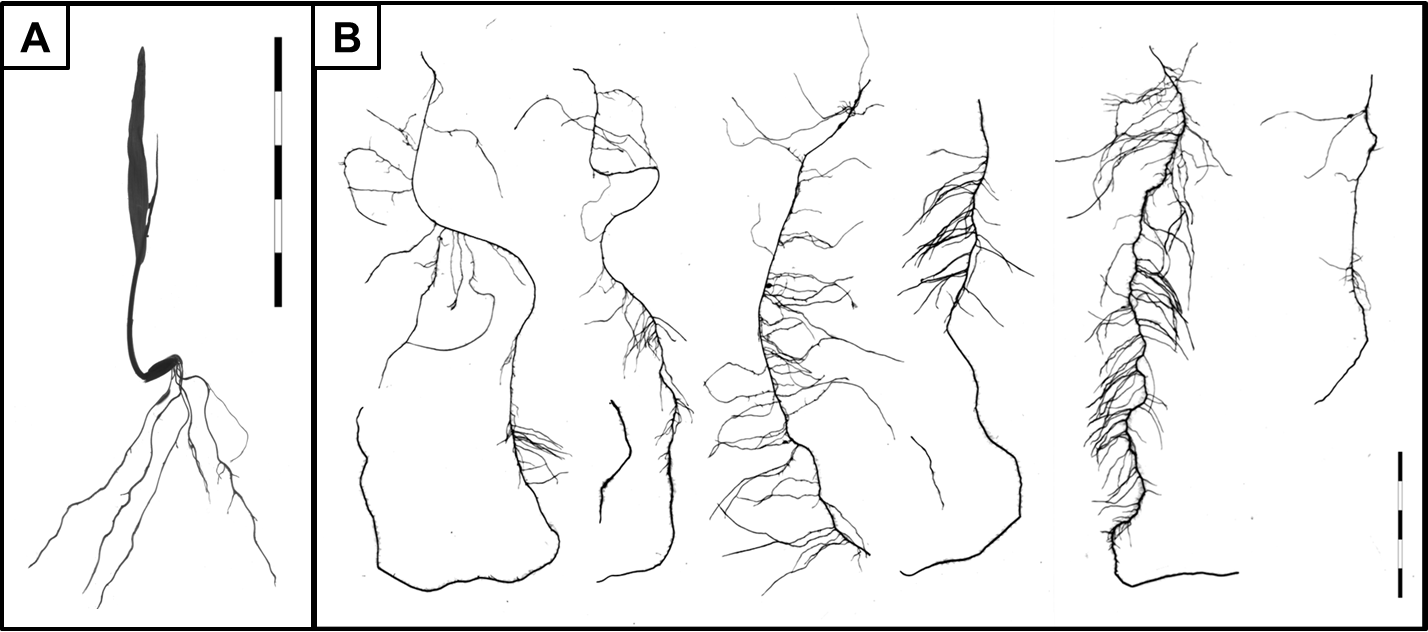

Supplement: Supplementary file 2 — 10.1186/s13007-016-0135-5. The output of the scanning of the root systems. A. A six-day-old seedling of barley scanned as a whole. B. Complete root system of a 14-day-old barley seedling (cv. Sebastian). [file 13007_2016_135_MOESM2_ESM.png]

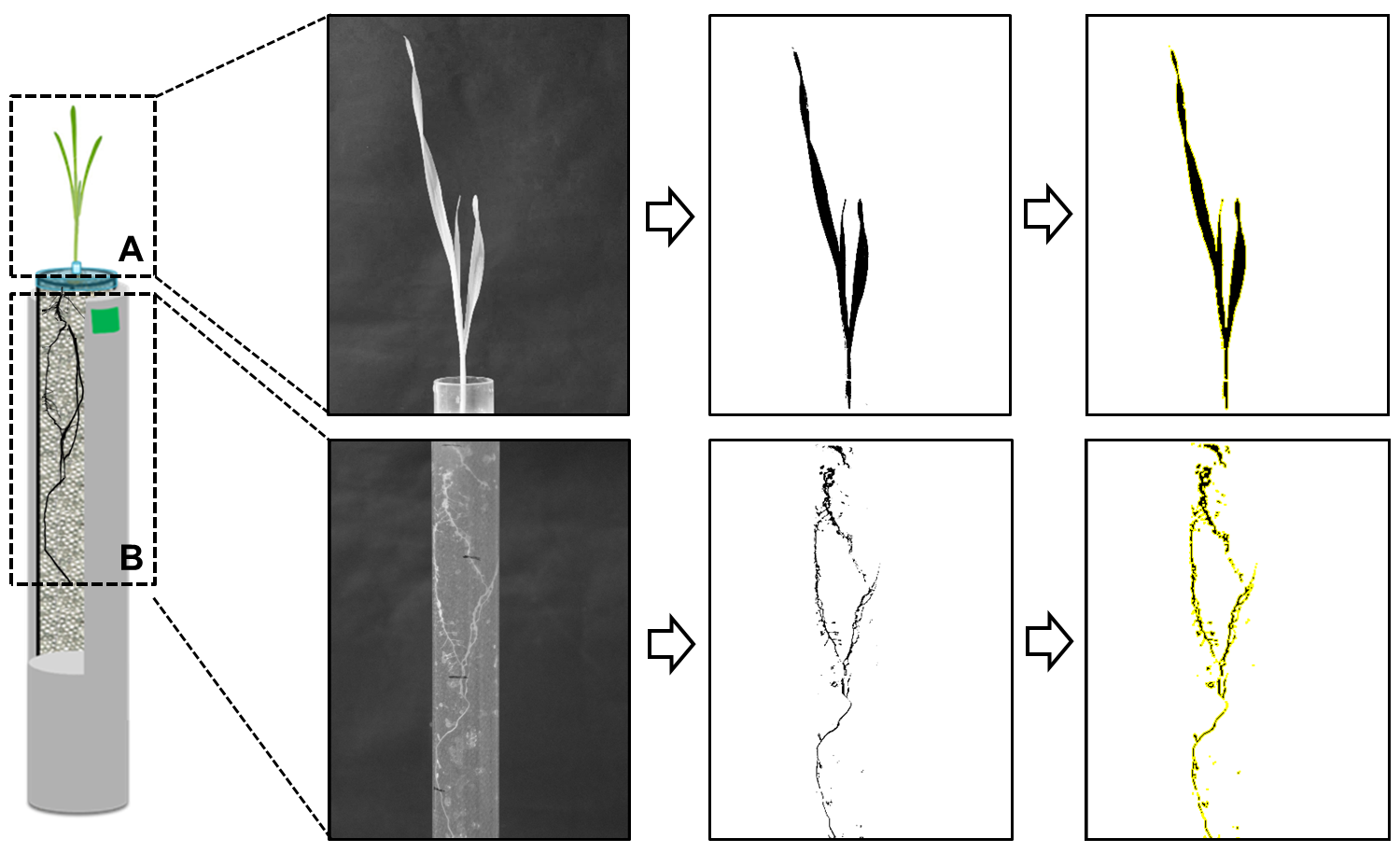

Supplement: Supplementary file 3 — 10.1186/s13007-016-0135-5 Illustration of the outcome of the image segmentation process of a barley seedling grown in the system. Shoot (A) and root (B) photographs were taken with a digital camera against a dark homogeneous background. [file 13007_2016_135_MOESM3_ESM.png]

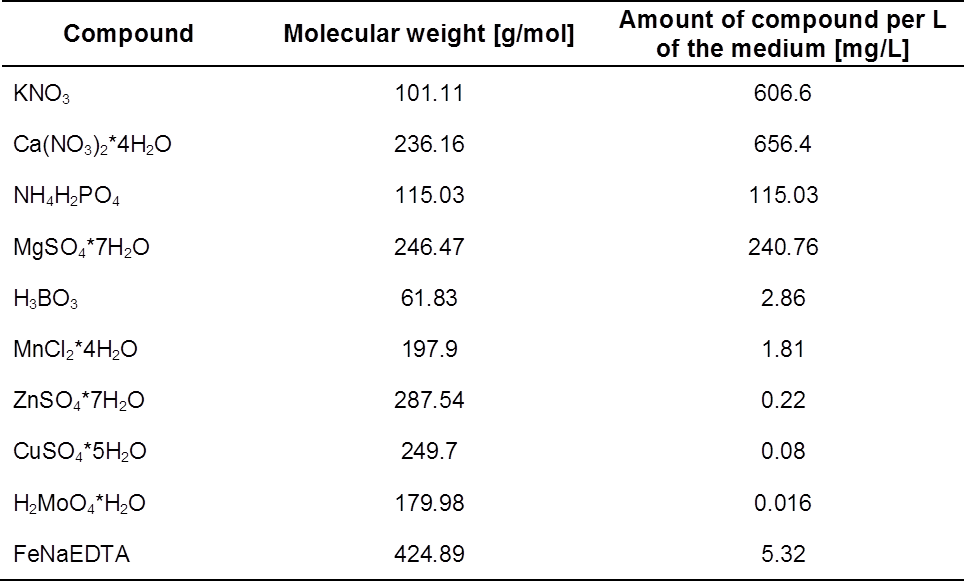

Supplement: Supplementary file 4 — 10.1186/s13007-016-0135-5 Amount of compounds used for the preparation of Hoagland’s medium No. 2 Basal Salt Mixture. The pH of the medium was adjusted to ~ 5,9-6,1° using 1 N NaOH. [file 13007_2016_135_MOESM4_ESM.png]
